# Retinoic acid-inducible gene-I like receptor pathway in cancer: modification and treatment

**DOI:** 10.3389/fimmu.2023.1227041

**Published:** 2023-08-16

**Authors:** Guangyuan Du, Zherui Xing, Jue Zhou, Can Cui, Chenyuan Liu, Yiping Liu, Zheng Li

**Affiliations:** ^1^ NHC Key Laboratory of Carcinogenesis, National Clinical Research Center for Geriatric Disorders, Key Laboratory of Carcinogenesis, Chinese Ministry of Health, Department of Oncology, Xiangya Hospital, Central South University, Changsha, Hunan, China; ^2^ Department of Clinical Medicine, Xingya School of Medicine of Central South University, Changsha, China; ^3^ Cancer Research Institute, School of Basic Medical Science, Central South University, Changsha, Hunan, China

**Keywords:** cancer, retinoic acid-inducible gene-I (RIG-I) like receptor pathway, epigenetic modulation, post-transcriptional modification, posttranslational modification, targeted therapy

## Abstract

Retinoic acid-inducible gene-I (RIG-I) like receptor (RLR) pathway is one of the most significant pathways supervising aberrant RNA in cells. In predominant conditions, the RLR pathway initiates anti-infection function via activating inflammatory effects, while recently it is discovered to be involved in cancer development as well, acting as a virus-mimicry responder. On one hand, the product IFNs induces tumor elimination. On the other hand, the NF-κB pathway is activated which may lead to tumor progression. Emerging evidence demonstrates that a wide range of modifications are involved in regulating RLR pathways in cancer, which either boost tumor suppression effect or prompt tumor development. This review summarized current epigenetic modulations including DNA methylation, histone modification, and ncRNA interference, as well as post-transcriptional modification like m6A and A-to-I editing of the upstream ligand dsRNA in cancer cells. The post-translational modulations like phosphorylation and ubiquitylation of the pathway’s key components were also discussed. Ultimately, we provided an overview of the current therapeutic strategies targeting the RLR pathway in cancers.

## 1 Introduction

Pattern recognition receptors (PRRs) are representative immune receptors in innate immunity that detect foreign and harmful molecules, such as those from pathogens and damaged cells (1, 2). Retinoic acid-inducible gene-I (RIG-I) like receptors (RLRs) are kind of PRRs, whose family members currently found include RIG-I (encoded by gene *DDX58*), melanoma differentiation-related gene 5 (MDA5, encoded by gene *IFIH1*) and genetic and physiological laboratory 2 (LGP2, encoded by gene *DHX58*) (3). The ligands of the RLRs are generally characterized as double-stranded RNAs (dsRNAs). While RIG-I preferentially binds to short dsRNAs with 5´ triphosphate, MDA5 is inclined to bind to longer dsRNAs (4). Once the abnormal dsRNAs are recognized, MDA5 and RIG-I are recruited to the mitochondrial surface and interact with the CARD domain of adaptor molecules known as mitochondrial antiviral signaling proteins (MAVS). Next, the complex effector molecules like TANK binding kinase (TBK1) can activate interferon regulatory factors (IRFs) or NF-κB transcription factors (5, 6).

Innate antiviral immunity relies on the recognition of viral nucleic acids through RLRs by most cell types, which triggers an antiviral immune response (7, 8). Recently, the function of the RLRs to recognize other aberrant RNA from autologous cells including cancer cells is brought to the spotlight (9). In most types of cancers, the RLR pathway activation plays an anti-tumor role in an IFN-dependent manner. The secreted IFNs further augment the expression of the RLR pathway members, driving a feed-forward loop potential for tumor elimination (10). Except for DNA mutations of the pathway’s key members, the research achievements on various modification of both the dsRNA and the RLR pathway components in cancer progression are updated.

Epigenetic modification is a kind of covalent modification of nucleic acid sequence and histone protein without changing DNA sequence (11). It is primarily categorized as DNA methylation, histone modification, chromatin remodeling, and non-coding RNA (ncRNA) interference. Moreover, emerging post-transcriptional modifications classified as “RNA epigenetics” are found (12). M6A modification is the most common one, and another main type is A-to-I editing of RNA. Beyond that, there are multiple post-translational modifications, such as protein phosphorylation, ubiquitylation, methylation, etc., which influence the function of key members in RLR pathway. Here we review the regulatory effects of the various modifications on the dsRNA production and key members associated with RLR pathway activation in tumors development, and highlight the latest progress in the tumor immunotherapy related to the RLR pathway.

## 2 Epigenetic modifications and dsRNA regulation with RLR pathway activation

**Figure 1:**
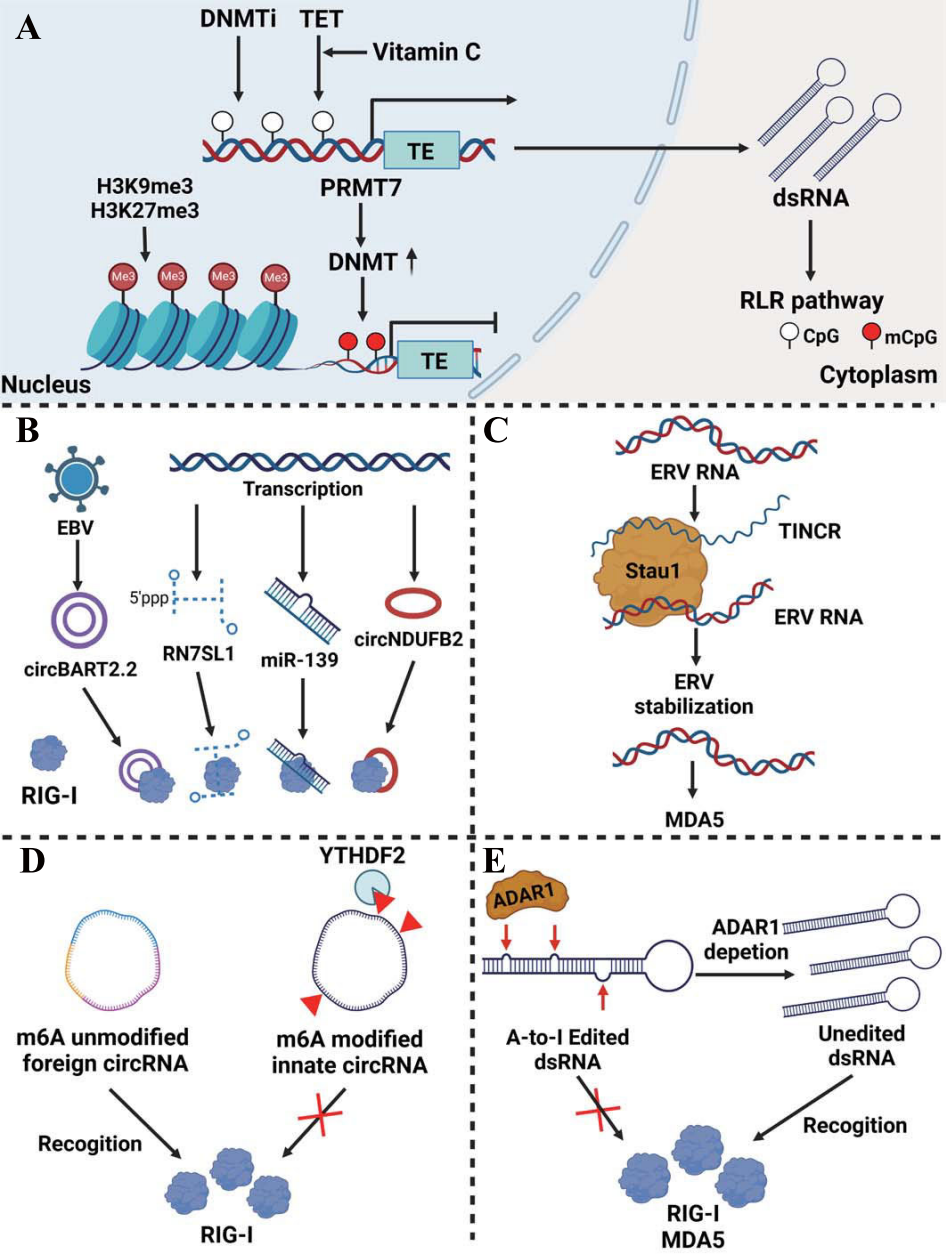
Epigenetic regulation of dsRNA production and modification in cancer. **(A)** Regulation of dsRNA expression at the DNA level. DNMTi induces endogenous dsRNA generation by reducing the DNA methylation level of TEs. TET enzymes cooperating with vitamin C actively convert 5-methylcytosine into 5-hydroxymethylcytosine in the LTR region of ERV which enhances dsRNA production. On the other hand, H3K9me3 and H3K27me3 histone modifications prohibit the transcription of the TEs. PRMT7 induced DNMT modifies the DNA methylation of the TEs, which abrogates the TEs transcription as well. **(B)** Non-coding RNA as dsRNA activates the RLR pathway. NcRNAs such as circBART2.2, RN7SL1, miR-139, and circNDUFB2 can act as dsRNAs recognized by RIG-I. **(C)** Stau1 stabilizes ERV RNA recognized by MDA5 via forming a lncRNA TINCR-Stau1-ERV complex. **(D)** Unmodified foreign circRNA, but not m6A-modified innate circRNA that is read by YTHDF2, directly activates RIG-I. **(E)** ADAR1 induces dsRNA A-to-I RNA editing thereby inhibiting the activation of the RLR pathway.

Tumor-derived endogenous dsRNAs are mainly produced by transposable elements (TEs) transcription, including long interspersed nuclear elements (LINEs), endogenous retroviruses (ERVs), and short interspersed nuclear elements (SINEs), etc (13). In addition, non-coding RNAs including miRNA, lncRNA and circRNA are presented as dsRNAs to active RLRs pathway in cancer (14). A multitude of epigenetic modifications ranging from the DNA to the RNA level have a significant impact on dsRNA generation and function including DNA methylation, histone methylation, m6A modification, and A-to-I RNA editing. Abnormal dsRNAs and their modification exert an obvious influence on the activation of the RLR pathway and tumor development (**Figure 1**).

### 2.1 DNA methylation and histone modification with dsRNA regulation

DNA methylation predominantly occurs on CpG sites in promoter region and serves to silence corresponding genes (15). SINEs, especially Alu retroelements, are the main source of DNA methyltransferase inhibitor (DNMTi)-induced endogenous dsRNA. Alu retroelements can form RNA stem–loops and become inverted repeated Alu (IR-Alu) functioning like dsRNA recognized by MDA5 (16). Indeed, DNMTi-mediated immune responses are mainly induced by the dsRNA increasing via DNA demethylation of the TEs to activate the RLR pathway, rather than by upregulation of DNMTi-induced viral defense genes. In colorectal cancer (CRC), low doses of 5-AZA-CdR (decitabine), a DNMTi, induces dsRNA formation and activates the RIG-I-MAVS pathway to produce anti-tumor immunity (17). It is also verified in ovarian cancer, mesothelioma, and acute myeloid leukemia (AML) that the increase in ERVs expression is induced by DNMTi which activates the RLR pathway and the interferon (IFN) response (18–20). Similarly, decitabine activates the expression of TEs, mainly including LINE1, ERV3-2, and ERV4700, which enhances the renal cell cancer response to immune checkpoint blockade (ICB) therapy (21).

In melanoma cells, inhibition of protein arginine methyltransferases 7 (PRMT7) decreases the expression of DNA methyltransferases and induces TEs transcription to generate pathologic dsRNA which are subsequently recognized by RIG-I and MDA5 (22). Interestingly, it is found in breast cancer cells and other cancers that the combination of vitamin C and decitabine greatly enhances the therapeutic effect of DNMTi. The mechanism may be that vitamin C can act as a cofactor for ten-eleven translocation (TET) enzymes to actively convert 5-methylcytosine into 5-hydroxymethylcytosine in the LTR region of ERVs and produce a synergistic effect, and the increased ERVs activates RIG-I and MDA5 (23).

Just like DNA methylation modifications, histone methylation and acetylation state also affect dsRNA expression. Through transforming the chromatin structure, some histone modifications promote gene expression, while others like H3K9me3 are associated with ‘closed’ and repressive heterochromatin (24). In taxane-resistant triple-negative breast cancer, the TEs transcription induced by hypomethylation is counteracted by histone H3K27me3 reprogram which prevents activation of the viral mimicry response and enhances tumor progression (25). Similarly, in prostate cancer cells, histone H3K9me3 modification abrogated TEs transcription and the subsequent RIG-I/MDA5-MAVS signaling, leading to the resistance of anti-androgen therapy (26). The combination treatment of DNMTi and histone deacetylase inhibitor (HDACi) is found to activate dsRNA which is sensed by MDA5 in ovarian cancer cells, thus exerting anti-tumor immunity affection via recruiting CD8 T and NK cells (27). The influence of DNA methylation and histone modification on dsRNA production was summarized in **Figure 1A**.

### 2.2 Non-coding RNAs as dsRNA activate RLR pathway

Non-coding RNAs have been shown to activate RLR signaling as dsRNA analogs (**Figure 1B**). RN7SL1(7SL) is a conserved, highly structured non-coding RNA that is present in all cell types. Normally it can bind to RNA binding protein SRP9/14 which protects it from recognition by RNA sensors. However, in breast cancer stromal cells, NOTCH-MYC signaling enhances RN7SL1 transcription, breaking the balance between it and SRP9/14. Unshielded RN7SL1 is packaged in exosomes and secreted which is recognized as virus-like RNA and eventually activates RIG-I-mediated inflammatory responses in cancer cells and promotes tumor progression (28). MiR-139 as an agonist induces RIG-I activation enhancing IFN-β production in prostate cancer (29). CircNDUFB2 emerges as a regulator of the RIG-I signaling pathway by decreasing the interaction between CARDs and the helicase domain of RIG-I and maintaining it in an active form. CircNDUFB2 is shown to be downregulated in non-small cell lung cancer (NSCLC) which leads to poor prognosis in patients (30). EBV-encoded CircBART2.2 can promote PD-L1 transcription and inhibit T cell function via binding the RIG-I helicase domain around nucleotides 114–165 in nasopharyngeal carcinoma, resulting in immune escape (31). LncRNA also plays an auxiliary role in the regulation of dsRNA stability (**Figure 1C**). In myelodysplastic syndromes (MDS) and AML, the dsRNA-binding protein Staufen1(Stau1) stabilized ERV RNA via forming a lncRNA TINCR-Stau1-ERV complex. The expression of Stau1 and TINCR negatively correlates with the outcome of DNMTi treatment (20).

### 2.3 RNA modification and dsRNA

M6A modification is modified via complexes of enzymes called ‘writers’, removed by ‘eraser’ proteins, and affects mRNA behavior via ‘reader’ proteins (32). M6A modification plays a role in the process of foreign circRNA activation of the RLR pathway. Unmodified foreign circRNA, but not m6A-modified innate circRNA that is read by YTHDF2 and directly activates RIG-I and downstream genes to induce innate immunity in melanoma (33) (**Figure 1D**). Additionally, some researches are being conducted on the enzyme adenosine deaminase acting on RNA (ADAR1), which has two isoforms in cells, including the constitutively expressing p110 isoform and the p150 isoform stimulated by activators like type I or type II IFN (34, 35). P150 recognizes dsRNA and transforms deaminate adenosine (A) to inosine (I). Because inosine cannot pair with thymine (T), it results in an unstable dsRNA secondary structure and the following degradation (36, 37). In esophageal squamous cell carcinoma (ESCC) and CRC, ADAR1 modifies dsRNA and reduces their stability, thereby inhibiting the activation of the RLR pathway and alleviating anti-tumor immunity (16, 38). Interestingly, in melanoma, after the absence of ADAR1, A-to-I RNA editing of dsRNA induced by interferon is reduced, resulting in a large number of stably expressed dsRNAs activating RNA sensors such as MDA5, inducing tumor cell growth inhibition and overcoming PD-1 resistance (39) (**Figure 1E**).

In addition, the loss of ADAR1 also leads to the accumulation of left-handed Z-form RNAs(Z-RNAs), which are generated from ISG 3´UTRs that contain dsRNA-forming inverted SINEs. And Z-RNAs activate their sensor ZBP1, leading to RIPK3-MLKL-mediated necrosis and overcoming ICB therapy unresponsiveness in mouse models of melanoma (40). DDX3X interacts with ADAR1, and the dual depletion of DDX3X and ADAR1 in breast cancer cells synergistically leads to the accumulation of dsRNA (41). In CRC, ADAR1 reduces dsRNA production induced by DNMTi treatment, which prevents activation of the MDA5 receptor. Moreover, DNMTi treatment is found to stimulate ADAR1 transcription and A-to-I editing, leading to the destabilization of immunogenic IR-Alu dsRNA. Thereby consumption of ADAR1 can ensure the stability of dsRNA which enhances the efficacy of epigenetic therapy (16).

## 3 Modification of key members in RLR pathway

**Figure 2:**
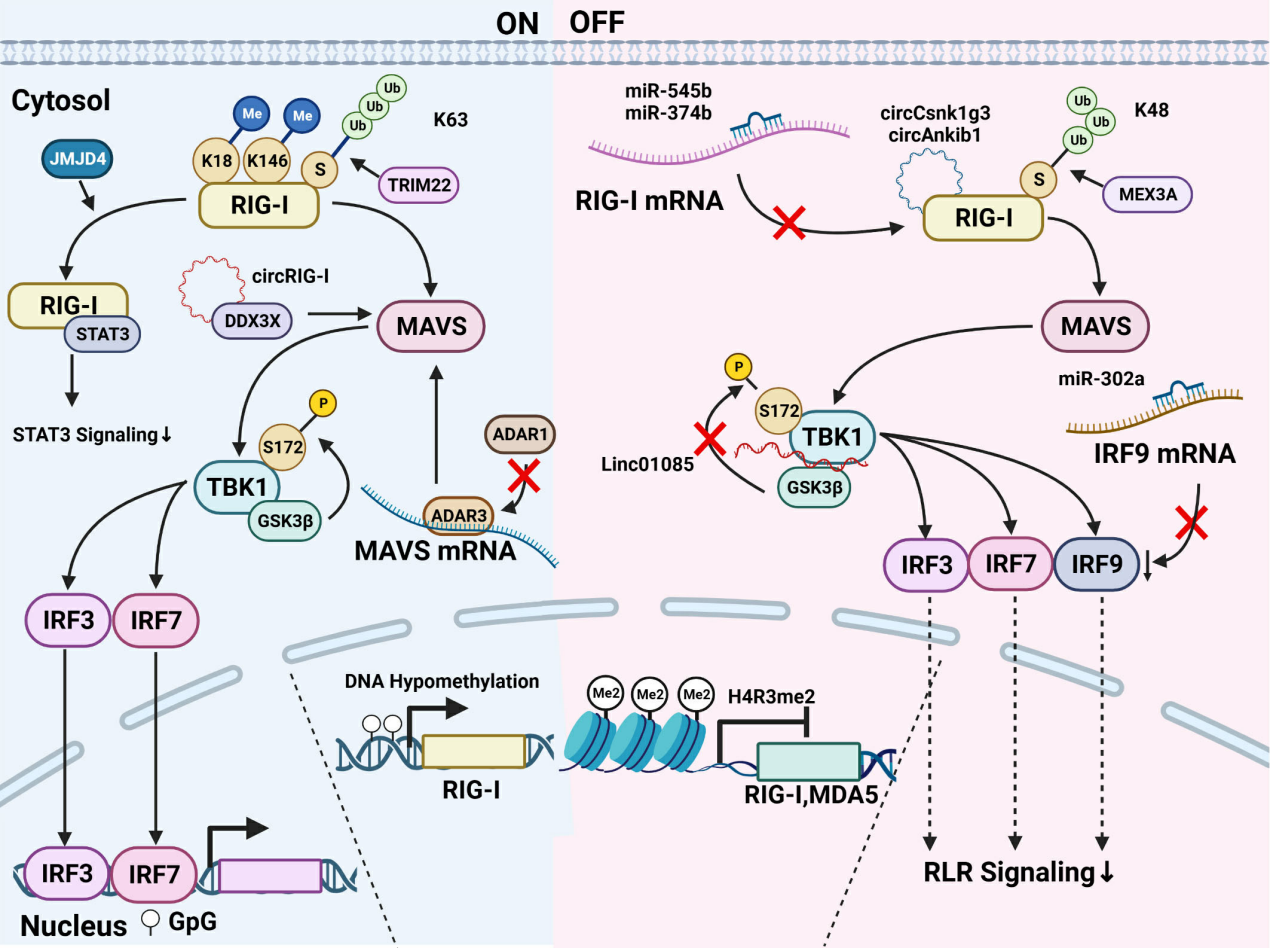
Modification of key members of the RLR pathway in cancer. Modifications of the RLR pathway can be divided into those that activate the RLR signaling and those that inhibit the pathway activation. (1) About the RLR signaling activation, DNA hypomethylation at the DNA level is reported to induce the RIG-I transcription and the farther activation of the pathway. At the protein level, K63-ubiquitylation modified by TRIM22 activates RIG-I. JMJD4 removes RIG-I mono-methylation at K18 and K146 and promotes its binding with STAT3, thus downregulating the STAT3 signaling. As for MAVS, ADAR3 competing with ADAR1 binds to the MAVS mRNA and upregulates MAVS protein level. The mutant RIG-I generates circRIG-I which binds to DDX3X and activates MAVS. TBK1 phosphorylated at Ser-172 by GSK3β activates the downstream signaling. (2) Modifications inhibiting the RLR pathway are summarized as follows. The histone H4R3me2 modification downregulates the transcription of RIG-I and MDA5. The expression of RIG-I is downregulated by miR-545 and miR-374b. CircRNA like circCsnk1g3 and circAnkib1 could directly interact with RIG-I protein and hampers the function of RIG-I. K48-linked ubiquitylation contributes to the degradation of RIG-I, and the E3 ubiquitin ligase MEX3A binds RIG-I to induce its degradation. LINC01085 abrogates the TBK1 interaction with GSK3β as well as the phosphorylation of TBK1 and inhibits the downstream of the pathway. The binding of miR-302a with IRF9 mRNA decreases the IRF9 level and the signaling transduction.

The modification of the key members in the RLR pathway has been widely explored, including DNA methylation, histone modification, chromatin accessibility, interference of ncRNAs, as well as protein modification like phosphorylation and ubiquitylation. As shown in **Figure 2**, those modifications are found to affect the activity of the key members of the RLR pathway in cancer cells, which further affects tumor development and therapy efficacy.

### 3.1 DNA modification and RNA regulation of key members in RLR pathway

A recent study shows that a decitabine can effectively hypomethylate *DDX58*/RIG-I promoter to arouse RIG-I-related innate immune response in Neuroblastoma (42). On the other hand, inhibition of PRMT7 enhances RIG-I and MDA5 expression via reduction of H4R3me2s repressive histone mark at the promoters, which boosts the expression of downstream targets such as interferon-stimulated genes(ISGs) in melanoma, thus hampering tumor growth (22).

Bioinformatics analysis shows that miR-193a-5p in lung cancer is of relevance to the RLR pathway and cancer pathway (43). MiR-545 targeting RIG-I mRNA is downregulated in oral squamous cell carcinoma (OSCC) and pancreatic ductal adenocarcinoma (PDAC), which plays as a tumor suppressor gene (44, 45). LncRNA FTX serves as the precursor of several functional miRNAs, such as miR-374b, miR-545, and miR-421. miR-545 derived from lncRNA FTX directly targets RIG-I and abrogates its expression, thus prompting the advancement of hepatocellular carcinoma (HCC) (46). The expression of RIG-I and PTEN is also downregulated by miR-545 and miR-374b in colorectal cancer, followed by the activation of PI3K-AKT oncogenic signaling that promotes colon cancer progression (47). Interestingly, a recent study demonstrates that the mRNA of mutant RIG-I generates circular RIG-I (circRIG-I). CircRIG-I activates innate immunity via DDX3X/MAVS/TRAF5/TBK1 axis and is upregulated in colon cancer (48).

In endometrial cancer, ADAR1 knockdown results in increased MDA5 and RIG-I expression (49). In glioblastoma (GBM), ADAR3 inhibits ADAR1-mediated editing in the MAVS 3’ UTR which induced upregulation of MAVS protein level without impacting MAVS mRNA expression (50). As for IRFs, it is demonstrated that miR-302a, which is regulated by ADAR1, could bind to the 3´UTR of IRF9 to attenuate its stability in gastric cancer (51). Therefore, it could be concluded that the modification of key members of the RLR pathway at the DNA and RNA level may have a significant influence on tumor progression, and the mechanism remains to be further explored.

### 3.2 Post-translational modification of key proteins in RLR pathway

The post-translational modification of RIG-I and MDA5 protein is very complex. It directly regulates the expression and activation of the proteins and subsequently the RLR pathway. A study lately demonstrates that circRNA like circCsnk1g3 and circAnkib1 could directly interact with RIG-I protein, which hampers the function of RIG-I. Silencing the two circRNAs in abemacilib-treated sarcoma cells induces a more significant level of interferon and pro-inflammatory factors than that in cells using a sole treatment of abemaciclib (52).

K63-linked ubiquitylation of RIG-I activates RIG-I and MDA5, while K48-linked ubiquitylation contributes to the degradation of RIG-I and MDA5, thereby influencing pathway activation (53). In lung adenocarcinoma, KEGG analysis shows that RBR E3 ubiquitin ligase is associated with the RIG-I-like pathway (54). In GBM, the E3 ubiquitin ligase MEX3A is strongly upregulated and binds RIG-I to induce its degradation. Conversely, the removal of MEX3A leads to an increase in RIG-I expression, which inhibits GBM growth (55). Moreover, it is recently reported that in GBM, RIG-I/NF-κB/CCAR1 axis is directly regulated by the TRIM22-NT5C2 complex. TRIM22 enhances the K63-linked ubiquitylation of RIG-I, whereas NT5C2 mediates K48-linked ubiquitylation (56).

Apart from ubiquitylation, ISGylation, a type of ubiquitin-like modification, is identified to be associated with the RLR pathway. Through bioinformatics analysis, ISG15, the ubiquitin-like modifier, was identified as a crucial gene associated with breast cancer development and metastasis via the RLR signaling pathway (57). In acute promyelocytic leukemia (APL), RIG-I together with STAT1 activates ISG-critical genes including ISG15, which is testified as a crucial factor to affect myeloid differentiation (58). In addition, it is recently reported that RIG-I is constitutively mono-methylated at K18 and K146, which is erased by demethylase JMJD4. Decreased RIG-I and highly expressed constitutively methylated RIG-I both prompt HCC cell proliferation, while JMJD4-demethylated RIG-I prevented the malignancy of HCC cells by downregulating STAT3 signaling (59).

In addition to RIG-I and MDA5, TBK1, the downstream effector of the RLRs, is also post-translationally regulated in cancers. In prostate cancer Docetaxel**-**resistant cells, low expression of LINC01085 enhances TBK1 interaction with GSK3β and accelerates phosphorylation of TBK1 at Ser-172, thereby increasing expression of PD-L1 and NF-κB (60). Taking together, various modifications of key proteins influencing the RLR signaling activation, only a few have been studied in cancer cells, thus requiring a deeper elucidation.

## 4 Targeting RLR pathway and cancer treatment

**Figure 3:**
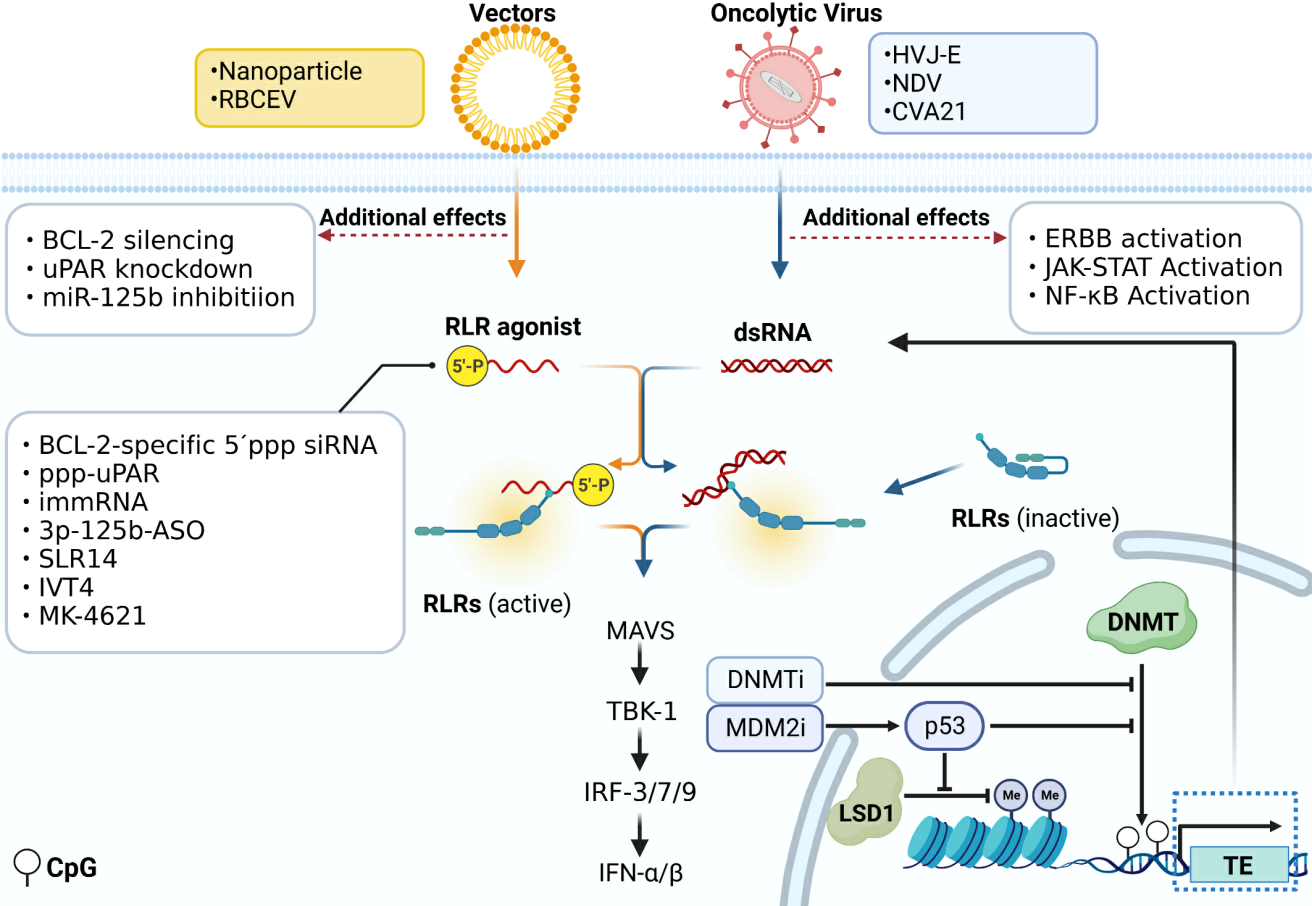
Immunotherapy directly acting on the RLR signal pathway. Various strategies targeting the RLR pathway in immunotherapy are exploited. DNA methylation inhibitors demethylate the DNA of endogenous TEs. It induces the formation of dsRNA and activates the RLR signaling cascade. P53 activated by MDM2 inhibitors could inhibit the function of DNMT and LSD1 and induce the ERVs expression which activates the downstream MAVS-IFN signaling. 5´ppp siRNA can work as a RIG-I ligand to be used in tumor treatment. The siRNA could be transfected by vectors like nanoparticles and RBCEV, and functions not only as ligands of RLRs, but also to silence the expression of other genes like BCL-2, uPAR, and miR-125b. Some modified viruses that act on RIG-I are used in tumor treatment, such as HVJ-E, NDV and CVA21. Some of them also activate ERBB, JAK-STAT and NF-κB signaling.

Various strategies targeting the RLR pathway in tumor therapy are exploited (**Figure 3**). Just as described before, DNA methylation inhibitors demethylate the DNA of endogenous TEs. It induces the formation of dsRNA and activates the RLR signaling cascade, further promoting IFN responses in cancer cells (18–20). 5,6-dihydro-5-azacytidine (DHAC), a reductive analog of decitabine, overcomes the disadvantage of hydrolytic instability resulting from saturated 5,6-double bonds, contributes to prolonged intravenous infusion time, and may avoid the acute toxicity caused by high-dose administration of decitabine (61, 62). It is reported that p53 activated by MDM2 inhibitors could inhibit the function of DNMT and LSD1, a histone demethylase, thus turning the tumor more immunogenic by inducing ERVs expression and activating the downstream MAVS-IFN signaling in melanoma (63).

Currently, some modified viruses that act on RIG-I are used in tumor treatment. In prostate cancer cells, the replication-incompetent hemagglutinating virus of Japanese envelope (HVJ-E) induces specific upregulation of pro-apoptotic factors downstream of the RIG-I/MAVS pathway like TNF-related apoptosis-inducing ligand (TRAIL) and Noxa, responsible for inducing cancer cell apoptosis (64). In melanoma, compared with IL-12 treatment alone, HVJ-E binding IL-12 significantly boosts the production of INF-γ by immune cells. This combination treatment results in more efficient eradication of melanoma and a reduction in the number of metastatic lesions (65). The Newcastle disease virus (NDV), classified as an oncolytic virus, exhibits a remarkable ability to eradicate diverse cancer cells selectively. The specificity ability can be attributed to the virus’s capacity to target multiple genes including ERBB, JAK-STAT, NF-κB, and RLR pathways (66). A novel ICAM-1–targeted immunotherapeutic-coxsackievirus A21 (CVA21) has finished the phase I trial. It gives rise to prominent inflammation in non-muscle-invasive bladder cancer(NMIBC) tissue through upregulation of RIG-I expression and IFN-inducible genes level (67).

As the RIG-I pathway can be activated by RNA containing 5´ triphosphate, 5´ppp siRNAs work as RIG-I ligands to be used in tumor treatment (68). A bifunctional strategy based on the combination of RLR pathway activation and targeting of the oncogene is studied in cancer treatment. BCL2-specific 5´ppp siRNA silences BCL2 expression and specifically activates the RLR pathway. This treatment method leads to melanoma cell apoptosis and enhances the amount of IFN-I to convert an immunosuppressive into an immune-supportive microenvironment (69). Nanoparticle Delivery of BCL2-specific 5´ppp siRNA also inhibits pancreatic cancer cell proliferation (70). Moreover, activating RIG-I itself sensitizes AML cells to BCL2 inhibitor drugs by remodeling mitochondrial metabolism (71). Two triphosphate-conjugated siRNAs that target uPAR (ppp-uPAR) are generated to knock down uPAR and simultaneously activate RIG-I. In melanoma, treatment with ppp-uPAR leads to the buildup of p53 and the activation of RIG-I-dependent proapoptotic signaling (72). The anti-cancer effects of two novel RIG-I agonists, namely the immunomodulatory RNA (immRNA) and anti-miR-125b-ASO with a 5´triphosphorylate modification (3p-125b-ASO), can be transported via extracellular vesicles derived from red blood cells (RBCEV). Both of them increase immune cell infiltration mediated by the activation of the RIG-I cascade and induce cell death in both mouse and human breast cancer cells (73). Stem-loop RNA (SLR)14 is a unique RIG-I agonist which delays tumor growth and prolongs the survival of mice with melanoma after intratumoral injection (74). IVT4, a RIG-I agonist, induces an IFN production and leads to the apoptosis of NSCLC cells (75).

## 5 Combination tumor therapy of drugs targeting RLR pathway with other treatments

Drugs that act on the RLR pathway can be combined with other immune therapy in tumor treatment. Chimeric antigen receptor (CAR)-T cell therapy has shown great potential in cancer treatment. CARs are artificially engineered receptors that can redirect lymphocytes to specific tumor cells expressing corresponding antigens (76). As previously reported that unshielded RN7SL1 is secreted by stromal cells in an autocrine loop, which activates the RLR pathway in cancer cells and accelerates tumor growth, metastasis, and therapeutic resistance (28). However, when RN7SL1 is delivered by CAR-T cells, immune cells in the tumor microenvironment are selectively transfected with RN7SL1 via extracellular vesicles which inhibits melanoma progression. In addition, an increase in CAR-T cell expansion and differentiation is also induced by RN7SL1, which enhances the treatment effector (77). Meanwhile, drugs targeting the RLR pathway can also be combined with anti-PD-1, anti-CTAL, and other immune checkpoint blockers to treat tumors. Using the syngeneic murine C1498 AML tumor model, Michael et al. find short 5´ppp RNA could activate the RLR pathway and induce the expression of programmed death ligand 1 (PD-L1) on AML cells, which enhances the effect of anti-PD-1 checkpoint blockade (78). Targeting RIG-I with 5´ppp RNA therapy can also effectively enhance the anti-CTLA-4 therapy effect. High RIG-I expression is significantly associated with durable clinical responses in melanoma patients treated with anti-CTLA-4 therapy (79). MK-4621, an oligonucleotide acting as a RIG-I agonist, passed phase I trials. It is proved to be safe with a modest antitumor activity both in monotherapy (NCT03065023) and in combination with anti-PD-1 therapy (NCT03739138) (80). The biodegradable poly (lactic-co-glycolic acid) (PLGA) particles that are “loaded” with tumor antigens and Riboxxim, a dsRNA adjuvant, activate murine and human dendritic cells by initiating the RLR and TLR pathways, which effectively suppress tumor development in multiple tumor models. Moreover, the therapeutic effect of cancer immunotherapy is further enhanced when combined with anti-CTLA-4 therapy (81).

Kinase inhibitors such as osimertinib play an anti-tumor role via repressing the function of key members of oncogenic pathways. It is reported that osimertinib pre-treatment sensitizes NSCLC cells to IVT4 treatment. The combination therapy induces a more significant tumor shrinkage than either treatment alone. It is also indicated that the combination therapy could maintain the PD-1 expression in tumor-infiltrating CD8 cells, while IVT4 treatment alone leads to a significant reduction of PD-1 expression (75). On the other hand, increased RIG-I impedes epidermal growth factor receptor-tyrosine kinase inhibitor (EGFR-TKI) treatment by activating IRF3. Loss of RIG-I enhances the sensitivity of EGFR mutant cells to erlotinib in NSCLC (82).

Immunotherapy targeting the RLR pathway exists in mutual interaction with chemotherapy and radiotherapy (RT). In nasopharyngeal carcinoma cells, upregulation of RIG-I renders the sensitiveness to paclitaxel via promoting IFN response and ER stress response-mediated apoptosis (83). In addition, in human myeloid leukemia, a 5´ppp siRNA targeting multi-drug resistance 1 (MDR1) simultaneously activates the RIR pathway, thus enhancing the anti-leukemia effect of doxorubicin (84). The activation of the RLR pathway can also promote the recovery of patients after chemotherapy and reduce the side effects of chemotherapy. During hematopoietic regeneration after chemotherapy, activating MDA5 generates an inflammatory response that is necessary for hematopoietic stem cells (HSCs) to exit quiescence and regenerate to replenish the hematopoietic system (85). Proper activation of the RLR pathway may be beneficial to improve the efficacy of RT. Radiation-induced DNA damage causes more cytosolic dsRNA formation, which subsequently activates the MDA5/MAVS/TBK1 pathway, leading to further tumor control in NSCLC and breast cancer (86). In addition, radiotherapy releases mitochondrial RNA(mtRNA) to the cytoplasm and thereby activates the RLR pathway (87). RIG-I interacts with X-ray repair cross complementing 4(XRCC4) which impedes DNA repair and sensitizes cancer cells to radiation therapy in lung adenocarcinoma (88). Diffusing alpha-emitting radiation therapy (DaRT) utilizes the diffusion of alpha-emitting atoms inside the tumor to activate tumor antigen recognition and induce a systemic antitumor immune response (89). Using various tumor mice models, a study reports that combination therapy of DaRT and RIG-I-like activation may reduce both tumor growth and distant metastases (90). It has been reported that overexpression of LGP2 may decrease the therapeutic effect of RT in GBM. RT induces overexpression of LGP2 which results in a significant decrease of IFN β expression and enhances the resistance of GBM to IR (91). However, in breast cancer after RT treatment, LGP2 is essential for the production of MDA5-mediated IFN-I in tumor dendritic cells (DCs). Using the MDA5/LGP2 agonist high molecular weight poly I:C could improve the antitumor effect of IR (92). The combination therapy of drugs that act on the RLR pathway and other tumor treatments are all summarized in **Table 1**.

**Table 1:** Combined tumor therapy related to drugs targeting RLR pathway.

Combined agents	RLR activation	Other therapy	Function	Ref
immune therapy	RN7SL1	CART	promote T cells expansion and inhibit tumor	(77)
	3pRNA	anti-PD-1, anti-CTLA	establish therapeutic sensitivity to immune checkpoint blockers	(78, 79)
	MK-4621	anti-PD-1	activate the RIG-I pathway	(80)
	Riboxxim packaging in PLGA with tumor antigens	anti-CTLA-4	initiate the RLR and TLR pathways and activate dendritic cells	(81)
targeted therapy	IVT4	Osimertinib	maintain the PD-1 expression in tumor-infiltrating CD8 cells	(75)
chemotherapy	RIG-I overexpression	Paclitaxel	inhibit chemoradiation resistance in nasopharyngeal carcinoma	(83)
	3p-siRNA-MDR1	Doxorubicin	involve RIG-I mediated IFN-I signal induction	(84)
	MDA5 activation	5-fluoruracil	promote the recovery of patients after chemotherapy	(85)
radiotherapy	RIG-I activation	Radiotherapy	impair DNA repair and sensitize cancer cells to RT	(88)
	PolyIC(PEI)	DaRT	reduce tumor growth and the spread of distant metastases	(90)
	LGP2 downregulation	Radiotherapy	increase the therapeutic effect of RT	(91)
	High molecular weight poly I:C	Radiotherapy	promote antitumor immunity of RT	(92)

## 6 Conclusion

The RLR pathway plays a vital role in antiviral immunity. In recent years, mounting studies have shown that it also plays an important role in tumor immunomodulatory. The RLR pathway members and upstream ligands are not only endowed with extensive epigenetic regulation but also regulated in varied post-transcriptional and post-translational modifications. Based on the brief introduction of pathway regulator mechanisms, we summarize modifications of the RLR pathway at the DNA, RNA, and protein levels involving pathway activation or inhibition, and the influence of the regulation mechanism on tumorigenesis in various types of cancer.

Immunotherapy against the RLR pathway is gradually carried out and can be divided into immunotherapy that acts directly on the RLR pathway and comprehensive therapy in combination with other treatments. The main targets of immunotherapy directed to the RLR pathway are RIG-I and MDA5, whose activation promotes anti-tumor immune environment formation. Combined therapeutic measures include the combination with immunotherapies like CAR-T and immune checkpoint blockers to improve the immunotherapeutic effect; the combination with chemotherapy to enhance the efficacy and reduce toxic side effects and the combination with radiotherapy to enhance the efficacy of radiotherapy. In addition, the design and optimization of RLR pathway activators can further improve the specificity of drugs to tumors and enhance combination therapy affection. Therefore, how to target and efficiently activate the internal RLR pathway in tumor cells and how to efficiently combine with other anti-tumor means are the research directions of targeted drugs in the future.

## Author contributions

All authors made substantial, direct, and intellectual contributions to the review. Under the direction of the corresponding authors, GD organized and wrote this review, ZX collected data, and others provided editorial assistance. The authors read and approved the final manuscript.

## Glossary

**Table:**

RIG-I	Retinoic acid-inducible gene-I
RLR	Retinoic acid-inducible gene-I like receptor
PRRs	Pattern recognition receptors
MDA5	Melanoma differentiation-related gene 5
LGP2	Genetic and physiological laboratory 2
dsRNA	Double-stranded RNA
MAVS	Mitochondrial antiviral signaling proteins
TBK1	TANK binding kinase
IRFs	Interferon regulatory factors
miR	MicroRNA
lncRNA	Long non-coding RNA
UTRs	Untranslated regions
TEs	Transposable elements
LINEs	Long interspersed nuclear elements
ERVs	Endogenous retroviruses
SINEs	Short interspersed nuclear elements
DNMTi	DNA methyltransferase inhibitor
IR-Alu	Inverted repeated Alu
CRC	Colorectal cancer
AML	Acute myeloid leukemia
IFN	Interferon
ICB	Immune checkpoint blockade
PRMT7	Protein arginine methyltransferases 7
TET	Ten-eleven translocation
HDACi	Histone deacetylase inhibitor
7SL	RN7SL1
MDS	Myelodysplastic syndromes
Stau1	Staufen1
NSCLC	Non-small cell lung cancer
ADAR1	Adenosine deaminase acting on RNA1
A	Adenosine
I	Inosine
T	Thymine
ESCC	Esophageal squamous cell carcinoma
Z-RNA	Z-form RNA
ISGs	Interferon-stimulated genes
OSCC	Oral squamous cell carcinoma
PDAC	Pancreatic ductal adenocarcinoma
HCC	Hepatocellular carcinoma
CircRIG-I	Circular RIG-I
GBM	Glioblastoma
APL	Acute promyelocytic leukemia
DHAC	5,6-dihydro-5-azacytidine
HVJ-E	Hemagglutinating virus of Japanese envelope
TRAIL	TNF-related apoptosis-inducing ligand
NDV	Newcastle disease virus
CVA21	Coxsackievirus A21
NMIBC	Non-muscle-invasive bladder cancer
ppp-uPAR	Triphosphate-conjugated siRNAs that target uPAR
immRNA	Immunomodulatory RNA
3p-125b-ASO	Anti-miR-125b ASO with a 5′
RBCEV	Extracellular vesicles derived from red blood cells
SLR	Stem-loop RNA
CAR	Chimeric antigen receptor
PD-L1	Programmed death ligand 1
EGFR-TKI	Epidermal growth factor receptor-tyrosine kinase inhibitor
RT	Radiotherapy
MDR1	Multi-drug resistance 1
HSCs	Hematopoietic stem cells
MtRNA	Mitochondrial RNA
XRCC4	X-ray repair cross complementing 4
DaRT	Diffusing alpha-emitting radiation therapy
DCs	Dendritic cells
PLGA	Biodegradable poly(lactic-co-glycolic acid)
TLR	Toll-like receptor

## References

[1] Mortaz E Adcock IM Tabarsi P Darazam IA Movassaghi M Garssen J. Pattern recognitions receptors in immunodeficiency disorders. Eur J Pharmacol (2017) 808:49–56. doi: 10.1016/j.ejphar.2017.01.014 28095323

[2] Gajewski TF Schreiber H Fu YX. Innate and adaptive immune cells in the tumor microenvironment. Nat Immunol (2013) 14(10):1014–22. doi: 10.1038/ni.2703 PMC4118725 24048123

[3] Barral PM Sarkar D Su ZZ Barber GN DeSalle R Racaniello VR. Functions of the cytoplasmic RNA sensors RIG-I and MDA-5: key regulators of innate immunity. Pharmacol Ther (2009) 124(2):219–34. doi: 10.1016/j.pharmthera.2009.06.012 PMC3165056 19615405

[4] Dias Junior AG Sampaio NG Rehwinkel J. A balancing act: MDA5 in antiviral immunity and autoinflammation. Trends Microbiol (2019) 27(1):75–85. doi: 10.1016/j.tim.2018.08.007 30201512 PMC6319154

[5] Seth R Sun L Ea C Chen ZJC. Identification and characterization of MAVS, a mitochondrial antiviral signaling protein that activates NF-kappaB and IRF 3. Cell (2005) 122(5):669–82. doi: 10.1016/j.cell.2005.08.012 16125763

[6] Cai H Yan L Liu N Xu M Cai H. IFI16 promotes cervical cancer progression by upregulating PD-L1 in immunomicroenvironment through STING-TBK1-NF-kB pathway. BioMed Pharmacother (2020) 123:109790. doi: 10.1016/j.biopha.2019.109790 31896065

[7] Rehwinkel J Gack MU. RIG-I-like receptors: their regulation and roles in RNA sensing. Nat Rev Immunol (2020) 20(9):537–51. doi: 10.1038/s41577-020-0288-3 PMC7094958 32203325

[8] Yoneyama M Onomoto K Jogi M Akaboshi T Fujita T. Viral RNA detection by RIG-I-like receptors. Curr Opin Immunol (2015) 32:48–53. doi: 10.1016/j.coi.2014.12.012 25594890

[9] Wu Y Wu X Wu L Wang X Liu Z. The anticancer functions of RIG-I-like receptors, RIG-I and MDA5, and their applications in cancer therapy. Transl Res (2017) 190:51–60. doi: 10.1016/j.trsl.2017.08.004 28917654

[10] Elion DL Cook RS. Harnessing RIG-I and intrinsic immunity in the tumor microenvironment for therapeutic cancer treatment. Oncotarget (2018) 9(48):29007–17. doi: 10.18632/oncotarget.25626 PMC6034747 29989043

[11] Dawson M Kouzarides TJC. Cancer epigenetics: from mechanism to therapy. Cell (2012) 150(1):12–27. doi: 10.1016/j.cell.2012.06.013 22770212

[12] Zhao LY Song J Liu Y Song CX Yi C. Mapping the epigenetic modifications of DNA and RNA. Protein Cell (2020) 11(11):792–808. doi: 10.1007/s13238-020-00733-7 32440736 PMC7647981

[13] Lanciano S Cristofari G. Measuring and interpreting transposable element expression. Nat Rev Genet (2020) 21(12):721–36. doi: 10.1038/s41576-020-0251-y 32576954

[14] Panni S Lovering RC Porras P Orchard S. Non-coding RNA regulatory networks. Biochim Biophys Acta Gene Regul Mech (2020) 1863(6):194417. 31493559 10.1016/j.bbagrm.2019.194417

[15] Moore LD Le T Fan G. DNA methylation and its basic function. Neuropsychopharmacology (2013) 38(1):23–38. doi: 10.1038/npp.2012.112 22781841 PMC3521964

[16] Mehdipour P Marhon S Ettayebi I Chakravarthy A Hosseini A Wang Y. Epigenetic therapy induces transcription of inverted SINEs and ADAR1 dependency. Nature (2020) 588(7836):169–73. doi: 10.1038/s41586-020-2844-1 33087935

[17] Roulois D Loo Yau H Singhania R Wang Y Danesh A Shen S. DNA-demethylating agents target colorectal cancer cells by inducing viral mimicry by endogenous transcripts. Cell (2015) 162(5):961–73. doi: 10.1016/j.cell.2015.07.056 PMC4843502 26317465

[18] Chiappinelli K Strissel P Desrichard A Li H Henke C Akman B. Inhibiting DNA Methylation Causes an Interferon Response in Cancer via dsRNA Including Endogenous Retroviruses. Cell (2015) 162(5):974–86. doi: 10.1016/j.cell.2015.07.011 PMC4556003 26317466

[19] Sun S Frontini F Qi W Hariharan A Ronner M Wipplinger M. Endogenous retrovirus expression activates type-I interferon signaling in an experimental mouse model of mesothelioma development. Cancer Lett (2021) 507:26–38. doi: 10.1016/j.canlet.2021.03.004 33713739

[20] Ku Y Park J Cho R Lee Y Park H Kim M. Noncanonical immune response to the inhibition of DNA methylation by Staufen1 via stabilization of endogenous retrovirus RNAs. Proc Natl Acad Sci U States America (2021) 118(13). doi: 10.1073/pnas.2016289118 PMC8020767 33762305

[21] de Cubas A Dunker W Zaninovich A Hongo R Bhatia A Panda A. DNA hypomethylation promotes transposable element expression and activation of immune signaling in renal cell cancer. JCI Insight (2020) 5(11). doi: 10.1172/jci.insight.137569 PMC7308050 32493845

[22] Srour N Villarreal O Hardikar S Yu Z Preston S Miller W. PRMT7 ablation stimulates anti-tumor immunity and sensitizes melanoma to immune checkpoint blockade. Cell Rep (2022) 38(13):110582. doi: 10.1016/j.celrep.2022.110582 35354055 PMC9838175

[23] Liu M Ohtani H Zhou W Ørskov A Charlet J Zhang Y. Vitamin C increases viral mimicry induced by 5-aza-2'-deoxycytidine. Proc Natl Acad Sci U States A (2016) 113(37):10238–44. doi: 10.1073/pnas.1612262113 PMC5027469 27573823

[24] Bannister AJ Kouzarides T. Regulation of chromatin by histone modifications. Cell Res (2011) 21(3):381–95. doi: 10.1038/cr.2011.22 PMC3193420 21321607

[25] Deblois G Tonekaboni S Grillo G Martinez C Kao Y Tai F. Epigenetic switch-induced viral mimicry evasion in chemotherapy-resistant breast cancer. Cancer Discov (2020) 10(9):1312–29. doi: 10.1158/2159-8290.CD-19-1493 32546577

[26] Baratchian M Tiwari R Khalighi S Chakravarthy A Yuan W Berk M. H3K9 methylation drives resistance to androgen receptor-antagonist therapy in prostate cancer. Proc Natl Acad Sci U States A (2022) 119(21):e2114324119. doi: 10.1073/pnas.2114324119 PMC9173765 35584120

[27] McDonald J Diab N Arthofer E Hadley M Kanholm T Rentia U. TP53Epigenetic therapies in ovarian cancer alter repetitive element expression in a -dependent manner. Cancer Res (2021) 81(20):5176–89. doi: 10.1158/0008-5472.CAN-20-4243 PMC8530980 34433584

[28] Nabet B Qiu Y Shabason J Wu T Yoon T Kim B. Exosome RNA unshielding couples stromal activation to pattern recognition receptor signaling in cancer. Cell (2017) 170(2):352–66.e13. doi: 10.1016/j.cell.2017.06.031 28709002 PMC6611169

[29] Nam RK Benatar T Amemiya Y Seth A. MiR-139 induces an interferon-beta response in prostate cancer cells by binding to RIG-1. Cancer Genomics Proteom (2021) 18(3):197–206. doi: 10.21873/cgp.20252 PMC8126337 33893074

[30] Li B Zhu L Lu C Wang C Wang H Jin H. circNDUFB2 inhibits non-small cell lung cancer progression via destabilizing IGF2BPs and activating anti-tumor immunity. Nat Commun (2021) 12(1):295. doi: 10.1038/s41467-020-20527-z 33436560 PMC7804955

[31] Ge J Wang J Xiong F Jiang X Zhu K Wang Y. Epstein-Barr virus-encoded circular RNA CircBART2.2 promotes immune escape of nasopharyngeal carcinoma by regulating PD-L1. Cancer Res (2021) 81(19):5074–88. doi: 10.1158/0008-5472.CAN-20-4321 PMC8974435 34321242

[32] Kan RL Chen J Sallam T. Crosstalk between epitranscriptomic and epigenetic mechanisms in gene regulation. Trends Genet (2022) 38(2):182–93. doi: 10.1016/j.tig.2021.06.014 PMC9093201 34294427

[33] Chen Y Chen R Ahmad S Verma R Kasturi S Amaya L. N6-methyladenosine modification controls circular RNA immunity. Mol Cell (2019) 76(1):96–109.e9. doi: 10.1016/j.molcel.2019.07.016 31474572 PMC6778039

[34] Li L Qian G Zuo Y Yuan Y Cheng Q Guo T. Ubiquitin-dependent turnover of adenosine deaminase acting on RNA 1 (ADAR1) is required for efficient antiviral activity of type I interferon. J Biol Chem (2016) 291(48):24974–85. doi: 10.1074/jbc.M116.737098 PMC5122768 27729454

[35] Rice GI Kasher PR Forte GM Mannion NM Greenwood SM Szynkiewicz M. Mutations in ADAR1 cause Aicardi-Goutieres syndrome associated with a type I interferon signature. Nat Genet (2012) 44(11):1243–8. doi: 10.1038/ng.2414 PMC4154508 23001123

[36] Liu J Wang F Zhang Y Liu J Zhao B. ADAR1-mediated RNA editing and its role in cancer. Front Cell Dev Biol (2022) 10:956649. doi: 10.3389/fcell.2022.956649 35898396 PMC9309331

[37] Nishikura K. Functions and regulation of RNA editing by ADAR deaminases. Annu Rev Biochem (2010) 79:321–49. doi: 10.1146/annurev-biochem-060208-105251 PMC2953425 20192758

[38] Wu Z Zhou J Zhang X Zhang Z Xie Y Liu J. Reprogramming of the esophageal squamous carcinoma epigenome by SOX2 promotes ADAR1 dependence. Nat Genet (2021) 53(6):881–94. doi: 10.1038/s41588-021-00859-2 PMC9124436 33972779

[39] Ishizuka J Manguso R Cheruiyot C Bi K Panda A Iracheta-Vellve A. Loss of ADAR1 in tumours overcomes resistance to immune checkpoint blockade. Nature (2019) 565(7737):43–8. doi: 10.1038/s41586-018-0768-9 PMC7241251 30559380

[40] Zhang T Yin C Fedorov A Qiao L Bao H Beknazarov N. ADAR1 masks the cancer immunotherapeutic promise of ZBP1-driven necroptosis. Nature (2022) 606(7914):594–602. doi: 10.1038/s41586-022-04753-7 35614224 PMC9373927

[41] Choi H Kwon J Cho MS Sun Y Zheng X Wang J. Targeting DDX3X Triggers Antitumor Immunity via a dsRNA-Mediated Tumor-Intrinsic Type I Interferon Response. Cancer Res (2021) 81(13):3607–20. doi: 10.1158/0008-5472.CAN-20-3790 PMC8597981 33941613

[42] Lin HY Chuang JH Wang PW Lin TK Wu MT Hsu WM. 5-aza-2'-deoxycytidine induces a RIG-I-related innate immune response by modulating mitochondria stress in neuroblastoma. Cells (2020) 9(9). doi: 10.3390/cells9091920 PMC7564572 32824929

[43] Xie ZC Tang RX Gao X Xie QN Lin JY Chen G. A meta-analysis and bioinformatics exploration of the diagnostic value and molecular mechanism of miR-193a-5p in lung cancer. Oncol Lett (2018) 16(4):4114–28. doi: 10.3892/ol.2018.9174 PMC6144214 30250529

[44] Song B Ji W Guo S Liu A Jing W Shao C. miR-545 inhibited pancreatic ductal adenocarcinoma growth by targeting RIG-I. FEBS Lett (2014) 588(23):4375–81. doi: 10.1016/j.febslet.2014.10.004 25315416

[45] Yuan G Wu H Du Y He F. Tumor suppressor role of microRNA-545 in oral squamous cell carcinoma. Oncol Lett (2019) 17(2):2063–8. doi: 10.3892/ol.2018.9820 PMC6341794 30675273

[46] Liu Z Dou C Yao B Xu M Ding L Wang Y. Ftx non coding RNA-derived miR-545 promotes cell proliferation by targeting RIG-I in hepatocellular carcinoma. Oncotarget (2016) 7(18):25350–65. doi: 10.18632/oncotarget.8129 PMC5041909 26992218

[47] Kwok ZH Zhang B Chew XH Chan JJ Teh V Yang H. Systematic Analysis of Intronic miRNAs Reveals Cooperativity within the Multicomponent FTX Locus to Promote Colon Cancer Development. Cancer Res (2021) 81(5):1308–20. doi: 10.1158/0008-5472.CAN-20-1406 33172934

[48] Song J Zhao W Zhang X Tian W Zhao X Ma L. Mutant RIG-I enhances cancer-related inflammation through activation of circRIG-I signaling. Nat Commun (2022) 13(1):7096. doi: 10.1038/s41467-022-34885-3 36402769 PMC9675819

[49] Nakamura K Shigeyasu K Okamoto K Matsuoka H Masuyama H. ADAR1 and AZIN1 RNA editing function as an oncogene and contributes to immortalization in endometrial cancer. Gynecol Oncol (2022) 166(2):326–33. doi: 10.1016/j.ygyno.2022.05.026 35697535

[50] Raghava Kurup R Oakes EK Manning AC Mukherjee P Vadlamani P Hundley HA. RNA binding by ADAR3 inhibits adenosine-to-inosine editing and promotes expression of immune response protein MAVS. J Biol Chem (2022) 298(9):102267. doi: 10.1016/j.jbc.2022.102267 35850307 PMC9418441

[51] Jiang L Park MJ Cho CJ Lee K Jung MK Pack CG. ADAR1 suppresses interferon signaling in gastric cancer cells by microRNA-302a-mediated IRF9/STAT1 regulation. Int J Mol Sci (2020) 21(17). doi: 10.3390/ijms21176195 PMC7504523 32867271

[52] Piras R Ko EY Barrett C De Simone M Lin X Broz MT. circCsnk1g3- and circAnkib1-regulated interferon responses in sarcoma promote tumorigenesis by shaping the immune microenvironment. Nat Commun (2022) 13(1):7243. doi: 10.1038/s41467-022-34872-8 36433954 PMC9700836

[53] Deng L Meng T Chen L Wei W Wang P. The role of ubiquitination in tumorigenesis and targeted drug discovery. Signal Transduct Target Ther (2020) 5(1):11. doi: 10.1038/s41392-020-0107-0 32296023 PMC7048745

[54] Ding H Wang Y Cui Y Chen Z Li Y Yang J. Comprehensive analysis of the expression and prognosis for RBR E3 ubiquitin ligases in lung adenocarcinoma. Thorac Cancer (2022) 13(17):2459–72. doi: 10.1111/1759-7714.14577 PMC9436683 35820682

[55] Bufalieri F Caimano M Lospinoso Severini L Basili I Paglia F Sampirisi L. The RNA-binding ubiquitin ligase MEX3A affects glioblastoma tumorigenesis by inducing ubiquitylation and degradation of RIG-I. Cancers (Basel) (2020) 12(2). doi: 10.3390/cancers12020321 PMC7072305 32019099

[56] Fei X Wu X Dou YN Sun K Guo Q Zhang L. TRIM22 orchestrates the proliferation of GBMs and the benefits of TMZ by coordinating the modification and degradation of RIG-I. Mol Ther Oncol (2022) 26:413–28. doi: 10.1016/j.omto.2022.08.007 PMC9465028 36159777

[57] Li Y Bai W Zhang L. The overexpression of CD80 and ISG15 are associated with the progression and metastasis of breast cancer by a meta-analysis integrating three microarray datasets. Pathol Oncol Res (2020) 26(1):443–52. doi: 10.1007/s12253-018-0478-5 30411299

[58] Wu SF Xia L Shi XD Dai YJ Zhang WN Zhao JM. RIG-I regulates myeloid differentiation by promoting TRIM25-mediated ISGylation. Proc Natl Acad Sci U S A (2020) 117(25):14395–404. doi: 10.1073/pnas.1918596117 PMC7322067 32513696

[59] Li Z Zhou Y Jia K Yang Y Zhang L Wang S. JMJD4-demethylated RIG-I prevents hepatic steatosis and carcinogenesis. J Hematol Oncol (2022) 15(1):161. doi: 10.2139/ssrn.4079097 36333807 PMC9636772

[60] Zhang J Li S Zhang J Zhang W Jiang J Wu H. Docetaxel resistance-derived LINC01085 contributes to the immunotherapy of hormone-independent prostate cancer by activating the STING/MAVS signaling pathway. Cancer Lett (2022) 545:215829. doi: 10.1016/j.canlet.2022.215829 35868534

[61] Beisler JA Abbasi MM Driscoll JS. Synthesis and antitumor activity of 5-azacytosine arabinoside. J Med Chem (1979) 22(10):1230–4. doi: 10.1021/jm00196a015 92567

[62] Curt GA Kelley JA Fine RL Huguenin PN Roth JS Batist G. A phase I and pharmacokinetic study of dihydro-5-azacytidine (NSC 264880). Cancer Res (1985) 45(7):3359–63. 2408749

[63] Zhou X Singh M Sanz Santos G Guerlavais V Carvajal LA Aivado M. Pharmacologic activation of p53 triggers viral mimicry response thereby abolishing tumor immune evasion and promoting antitumor immunity. Cancer Discovery (2021) 11(12):3090–105. doi: 10.1158/2159-8290.CD-20-1741 PMC9414294 34230007

[64] Matsushima-Miyagi T Hatano K Nomura M Li-Wen L Nishikawa T Saga K. TRAIL and Noxa are selectively upregulated in prostate cancer cells downstream of the RIG-I/MAVS signaling pathway by nonreplicating Sendai virus particles. Clin Cancer Res (2012) 18(22):6271–83. doi: 10.1158/1078-0432.CCR-12-1595 23014529

[65] Saga K Tamai K Yamazaki T Kaneda Y. Systemic administration of a novel immune-stimulatory pseudovirion suppresses lung metastatic melanoma by regionally enhancing IFN-γ production. Clin Cancer Res (2013) 19(3):668–79. doi: 10.1158/1078-0432.CCR-12-1947 23251005

[66] Chen Y Zhu S Pei Y Hu J Hu Z Liu X. Differential microRNA expression in newcastle disease virus-infected HeLa cells and its role in regulating virus replication. Front Oncol (2021) 11:616809. doi: 10.3389/fonc.2021.616809 34150610 PMC8211993

[67] Annels N Mansfield D Arif M Ballesteros-Merino C Simpson G Denyer M. Phase I trial of an ICAM-1-targeted immunotherapeutic-coxsackievirus A21 (CVA21) as an oncolytic agent against non muscle-invasive bladder cancer. Clin Cancer Res (2019) 25(19):5818–31. doi: 10.1158/1078-0432.CCR-18-4022 31273010

[68] Kato H Takeuchi O Sato S Yoneyama M Yamamoto M Matsui K. Differential roles of MDA5 and RIG-I helicases in the recognition of RNA viruses. Nature (2006) 441(7089):101–5. doi: 10.1038/nature04734 16625202

[69] Poeck H Besch R Maihoefer C Renn M Tormo D Morskaya S. 5'-Triphosphate-siRNA: turning gene silencing and Rig-I activation against melanoma. Nat Med (2008) 14(11):1256–63. doi: 10.1038/nm.1887 18978796

[70] Das M Shen L Liu Q Goodwin TJ Huang L. Nanoparticle delivery of RIG-I agonist enables effective and safe adjuvant therapy in pancreatic cancer. Mol Ther (2019) 27(3):507–17. doi: 10.1016/j.ymthe.2018.11.012 PMC6401191 30545600

[71] Fresquet V Garcia-Barchino M Larrayoz M Celay J Vicente C Fernandez-Galilea M. Endogenous retroelement activation by epigenetic therapy reverses the warburg effect and elicits mitochondrial-mediated cancer cell death. Cancer Discov (2021) 11(5):1268–85. doi: 10.1158/2159-8290.CD-20-1065 33355179

[72] Matheis F Heppt M Graf S Düwell P Kammerbauer C Aigner A. A bifunctional approach of immunostimulation and uPAR inhibition shows potent antitumor activity in melanoma. J Invest Dermatol (2016) 136(12):2475–84. doi: 10.1016/j.jid.2016.07.026 27498344

[73] Peng B Nguyen T Jayasinghe M Gao C Pham T Vu L. Robust delivery of RIG-I agonists using extracellular vesicles for anti-cancer immunotherapy. J Extracell Vesicles (2022) 11(4):e12187. doi: 10.1002/jev2.12187 35430766 PMC9013404

[74] Jiang X Muthusamy V Fedorova O Kong Y Kim D Bosenberg M. Intratumoral delivery of RIG-I agonist SLR14 induces robust antitumor responses. J Exp Med (2019) 216(12):2854–68. doi: 10.1084/jem.20190801 PMC6888973 31601678

[75] Brägelmann J Lorenz C Borchmann S Nishii K Wegner J Meder L. MAPK-pathway inhibition mediates inflammatory reprogramming and sensitizes tumors to targeted activation of innate immunity sensor RIG-I. Nat Commun (2021) 12(1):5505. doi: 10.1038/s41467-021-25728-8 34535668 PMC8448826

[76] Sterner RC Sterner RM. CAR-T cell therapy: current limitations and potential strategies. Blood Cancer J (2021) 11(4):69. doi: 10.1038/s41408-021-00459-7 33824268 PMC8024391

[77] Johnson L Lee D Eacret J Ye D June C Minn A. The immunostimulatory RNA RN7SL1 enables CAR-T cells to enhance autonomous and endogenous immune function. Cell (2021) 184(19):4981–95.e14. doi: 10.1016/j.cell.2021.08.004 34464586 PMC11338632

[78] Ruzicka M Koenig L Formisano S Boehmer D Vick B Heuer E. RIG-I-based immunotherapy enhances survival in preclinical AML models and sensitizes AML cells to checkpoint blockade. Leukemia (2020) 34(4):1017–26. doi: 10.1038/s41375-019-0639-x PMC7214254 31740809

[79] Heidegger S Wintges A Stritzke F Bek S Steiger K Koenig P. RIG-I activation is critical for responsiveness to checkpoint blockade. Sci Immunol (2019) 4(39). doi: 10.1126/sciimmunol.aau8943 31519811

[80] Moreno V Calvo E Middleton MR Barlesi F Gaudy-Marqueste C Italiano A. Treatment with a retinoic acid-inducible gene I (RIG-I) agonist as monotherapy and in combination with pembrolizumab in patients with advanced solid tumors: results from two phase 1 studies. Cancer Immunol Immunother (2022) 71(12):2985–98. doi: 10.1007/s00262-022-03191-8 PMC10991664 35596791

[81] Koerner J Horvath D Herrmann V MacKerracher A Gander B Yagita H. PLGA-particle vaccine carrying TLR3/RIG-I ligand Riboxxim synergizes with immune checkpoint blockade for effective anti-cancer immunotherapy. Nat Commun (2021) 12(1):2935. doi: 10.1038/s41467-021-23244-3 34006895 PMC8131648

[82] Gong K Guo G Panchani N Bender ME Gerber DE Minna JD. EGFR inhibition triggers an adaptive response by co-opting antiviral signaling pathways in lung cancer. Nat Cancer (2020) 1(4):394–409. doi: 10.1038/s43018-020-0048-0 33269343 PMC7706867

[83] Jing D Zhou W Shen L Zhang Q Xie WT Shen E. RIG-I promotes IFN/JAK2 expression and the endoplasmic reticulum stress response to inhibit chemoradiation resistance in nasopharyngeal carcinoma. Cancer Med (2019) 8(14):6344–57. doi: 10.1002/cam4.2501 PMC6797570 31464090

[84] Li D Gale R Liu Y Lei B Wang Y Diao D. 5'-Triphosphate siRNA targeting MDR1 reverses multi-drug resistance and activates RIG-I-induced immune-stimulatory and apoptotic effects against human myeloid leukaemia cells. Leuk Res (2017) 58:23–30. doi: 10.1016/j.leukres.2017.03.010 28380403

[85] Clapes T Polyzou A Prater P Sagar Morales-Hernández A Ferrarini M. Chemotherapy-induced transposable elements activate MDA5 to enhance haematopoietic regeneration. Nat Cell Biol (2021) 23(7):704–17. doi: 10.1038/s41556-021-00707-9 PMC8492473 34253898

[86] Lee A Pan D Bao X Hu M Li F Li C. Endogenous retrovirus activation as a key mechanism of anti-tumor immune response in radiotherapy. Radiat Res (2020) 193(4):305–17. doi: 10.1667/RADE-20-00013 PMC7359417 32074012

[87] Tigano M Vargas D Tremblay-Belzile S Fu Y Sfeir A. Nuclear sensing of breaks in mitochondrial DNA enhances immune surveillance. Nature (2021) 591(7850):477–81. doi: 10.1038/s41586-021-03269-w 33627873

[88] Guo G Gao M Gao X Zhu B Huang J Tu X. Reciprocal regulation of RIG-I and XRCC4 connects DNA repair with RIG-I immune signaling. Nat Commun (2021) 12(1):2187. doi: 10.1038/s41467-021-22484-7 33846346 PMC8041803

[89] DOmankevich V Cohen A Efrati M Schmidt M Rammensee HG Nair SS. Combining alpha radiation-based brachytherapy with immunomodulators promotes complete tumor regression in mice via tumor-specific long-term immune response. Cancer Immunol Immunother (2019) 68(12):1949–58. doi: 10.1007/s00262-019-02418-5 PMC6877484 31637474

[90] DOmankevich V Efrati M Schmidt M Glikson E Mansour F Shai A. RIG-1-like receptor activation synergizes with intratumoral alpha radiation to induce pancreatic tumor rejection, triple-negative breast metastases clearance, and antitumor immune memory in mice. Front Oncol (2020) 10:990. doi: 10.3389/fonc.2020.00990 32766128 PMC7379859

[91] Widau R Parekh A Ranck M Golden D Kumar K Sood R. RIG-I-like receptor LGP2 protects tumor cells from ionizing radiation. Proc Natl Acad Sci U States A (2014) 111(4):E484–91. doi: 10.1073/pnas.1323253111 PMC3910628 24434553

[92] Zheng W Ranoa D Huang X Hou Y Yang K Poli E. RIG-I-like receptor LGP2 is required for tumor control by radiotherapy. Cancer Res (2020) 80(24):5633–41. doi: 10.1158/0008-5472.CAN-20-2324 33087322

